# Regulation of autophagy by Rab27B in colorectal cancer

**DOI:** 10.1016/j.biocel.2024.106693

**Published:** 2024-11-13

**Authors:** Sahida Afroz, Ranjan Preet, Vikalp Vishwakarma, Andrew E. Evans, Alexa N. Magstadt, Dan A. Dixon

**Affiliations:** a Department of Molecular Biosciences, University of Kansas, Lawrence, KS, USA; b University of Kansas Cancer Center, Kansas City, KS, USA

**Keywords:** Rab27B, Colorectal cancer, Autophagy

## Abstract

Autophagy is a cellular recycling process that is associated with tumor growth, anti-tumor immune responses, and therapy resistance in colorectal cancer (CRC). In this report, we identify the small GTPase Rab27B to control the autophagy process in CRC. Depletion of Rab27B showed an abnormal accumulation of autophagy vesicles and increased autophagy markers in CRC cells, indicating autophagy dysregulation. Image analysis indicated that autophagy flux is blocked at the autophagosome/lysosome fusion step when Rab27B is lost. While Rab27B deficient cells are proficient at growth under 2D *in vitro* conditions, cell growth was significantly impacted in both *in vitro* 3D growth and *in vivo* tumorigenesis studies. Together, these results demonstrate a new role of Rab27B in the autophagy trafficking process in CRC and identify Rab27B as a potential therapeutic target for CRC.

## 1. Introduction

Approximately 4.6 % of men (1 in 22) and 4.2 % of women (1 in 24) will be diagnosed with colorectal cancer (CRC) in their lifetime, making it the second leading cause of cancer-related death in the US (Society, 2020). Although CRC death rates have decreased since 1975 due to screening, new treatments, and changes in risk factors, there was still an estimated 153,020 new cases of CRC and 52,550 associated deaths in 2023 (Siegel et al., 2023). Cancer cells consistently deal with various stress and growth challenges like nutrition deprivation, hypoxia, immune response, and anti-cancer treatments. They alter different cellular pathways to deal with these stresses to ensure survival and growth. One such pathway is autophagy, which is increasingly recognized to have a major role in cancer initiation, maintenance, and resistance to therapy (Chen and Debnath, 2010; Chen and Karantza-Wadsworth, 2009; Jin and White, 2007; Karantza-Wadsworth et al., 2007; Karantza-Wadsworth and White, 2007; Levine, 2006; Li et al., 2020; Mathew et al., 2007). Autophagy is kept at a basal level in all cells, which helps maintain cellular homeostasis and adapt to stress responses such as energy supply during starvation (Glick et al., 2010). However, in many cancers, autophagy is activated at a higher level and correlates with poor prognosis (Amaravadi et al., 2016; Giatromanolaki et al., 2011; Koustas et al., 2019). In particular, mutations in *KRAS*, an oncogene frequently mutated (30–50 %) in colorectal tumors, are associated with higher levels of autophagy (Alves et al., 2015; Guo et al., 2011). This heightened level of autophagy has been shown to promote tumorigenesis by promoting cell survival against various stressors occurring through nutrient depravation, hypoxia, and chemotherapy (Mahgoub et al., 2022; Mokarram et al., 2017; Sun et al., 2015). Autophagy also provides anti-tumor immune response against NK (natural killer) cells and resistance to cancer therapy (Baginska et al., 2013; Baginska et al., 2013; Chen et al., 2010; Su et al., 2016). Reports show that inhibition of autophagy re-sensitized resistant cancer cells to chemotherapy and radiation, leading to tumor infiltration of NK cells (Apel et al., 2008; Bellodi et al., 2009; Mgrditchian et al., 2017). As autophagy is being increasingly recognized as a potential target for cancer therapy, the only approved drug targeting autophagy in cancer is hydroxychloroquine (HCQ) (Chude and Amaravadi, 2017; Mahalingam et al., 2014). HCQ has been shown to disrupt the autophagic process by blocking the fusion of autophagosomes and lysosomes (Mauthe et al., 2018). However, HCQ has significant limitations, including impairment of necessary lysosomal function, a high concentration required for activity *in vivo*, and a long half-life contributing to side effects. Thus, the need for identifying new factors controlling autophagy in cancer as potential drug targets is warranted.

Given that autophagy is increased in cancer, the vesicle trafficking process related to autophagy is also regulated to accommodate this increased demand due to stress. Rab proteins are small GTPases that belong to the RAS-like GTPase superfamily and regulate different cellular vesicle trafficking processes (Bhuin and Roy, 2014; Hutagalung and Novick, 2011). Various Rab proteins have been shown to be involved in different stages of autophagy (Bento et al., 2013; Gutierrez et al., 2004; Lorincz et al., 2017). Rab1 contributes to the initiation of autophagy, Rab9 modulates the fusion of autophagosomes with late endosomes, Rab7 is implicated in the fusion of autophagosomes with lysosomes, whereas Rab11, Rab30, and Rab37 are essential for starvation-induced autophagy (Arakawa et al., 2017; Gutierrez et al., 2004; Longatti et al., 2012; Oda et al., 2016; Sheng et al., 2018; Zoppino et al., 2010). Rab27B has been demonstrated in endosomal trafficking, small extracellular vesicle (sEV)/exosome secretion, and membrane delivery of secretory granules (Gomi et al., 2007; Ostrowski et al., 2010). With regard to cancer, overexpression of Rab27B is observed, and associated with poor prognosis and metastasis in various cancers, including CRC (Bao et al., 2014; Koh and Song, 2019; Ren et al., 2016; Zhang et al., 2012; Zhao et al., 2016). Increased expression of Rab27B can promote cell growth both *in vitro* and *in vivo* in breast, pancreatic, colorectal, and lung cancer cells (Cheng et al., 2019; Hendrix et al., 2010; Li et al., 2017, 2018; Zhu et al., 2019). However, the mechanism (s) underlying the tumor-promoting ability of Rab27B are not well understood.

In this report, we have identified Rab27B to be overexpressed in CRC and play a critical role in controlling autophagy flux. Depletion of Rab27B in CRC cell lines blocks autophagy flux and results in an accumulation of autophagosomes, indicating that Rab27B plays a role in the fusion step between autophagosomes and lysosomes. Additionally, we show that Rab27B deletion inhibits CRC cell growth *in vitro* under stress-related conditions and under *in vivo* tumor growth conditions, underscoring Rab27B as a potential therapeutic target for autophagy inhibition in CRC.

## 2. Results

### 2.1. Rab27B is overexpressed in CRC

**Fig. 1.**
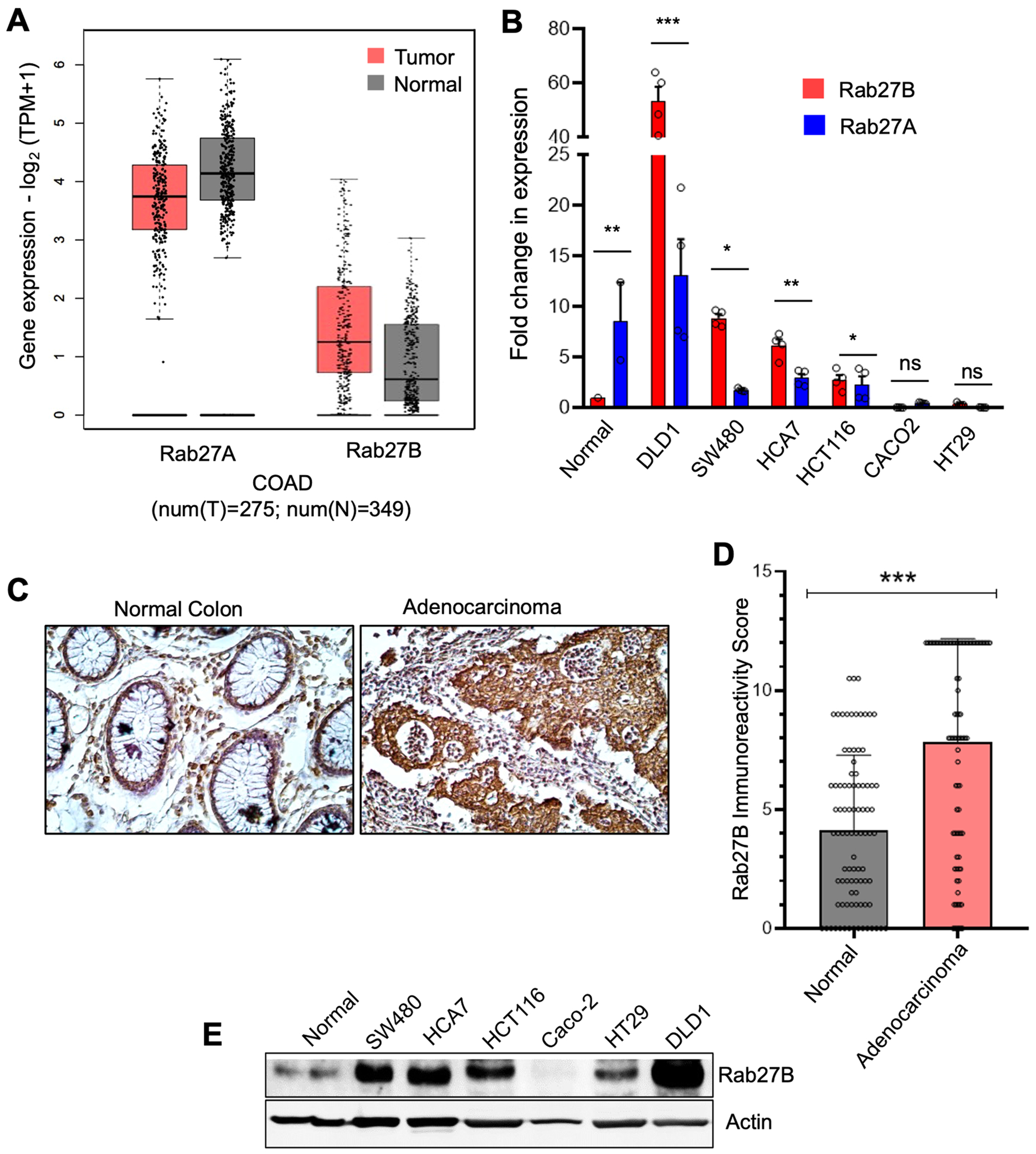
Rab27B is overexpressed in CRC. (A) Boxplot derived from gene expression data comparing Rab27A and Rab27B expression in normal colon tissue and colon adenocarcinoma (COAD) tissue (GEPIA: http://gepia.cancer-pku.cn/, Normal = 349, Tumor = 275.). (B) Analysis of Rab27A and Rab27B expression in normal colon tissue (n=4) and CRC cell lines (DLD1, SW480, HCA7, HCT116, HT29, and MOSER) by qPCR. All data presented are the average ± SD of four independent experimental repeats. (C) Representative immunohistochemistry images of colon adenocarcinoma and adjacent normal tissue from tissue microarray stained for Rab27B. (D) Plot showing immunoreactivity score (IRS) (n=90). (E) Western blot of Rab27B expression in normal tissue and CRC cell lines (SW480, HCA7, HCT116, Caco-2, HT29, and DLD1). Actin was used as the loading control. (*, p ≤ 0.05; **, p ≤ 0.01; ***, p ≤ 0.001).

We evaluated Rab27B expression status in CRC by analyzing both Rab27B mRNA and protein levels. Using GEPIA (Gene Expression Profiling Interactive Analysis) (Tang et al., 2017), Rab27B expression was compared between 275 CRC tumors and 349 normal colon tissue. Rab27B showed approximately 2-fold increased mRNA expression in tumors compared to normal tissues, whereas Rab27A expression was reduced in tumors (Fig. 1A). Similarly, Rab27B expression was elevated 3.5- to 45-fold in CRC cell lines (HCT116, HCA7, SW480, and DLD1), and Rab27A levels were decreased compared to normal colon tissue (Fig. 1B). Using tissue micro-arrays containing colon adenocarcinoma (n=90) and matched adjacent normal tissue, Rab27B protein expression was assayed by immunohistochemistry (IHC). Shown in Fig. 1C, most tumor sections (81) were positive for Rab27B expression with elevated cytoplasmic staining. Tumor sections showed a higher immunoreactivity score (IRS) score compared to adjacent normal tissue (Fig. 1D). Rab27B protein expression in CRC cell lines was assayed by western blotting. Consistent with mRNA expression, Rab27B protein was increased in CRC cell lines HCT116, HCA7, SW480, and DLD1 compared to normal tissue (Fig. 1E). These results demonstrate Rab27B to be overexpressed in CRC, whereas expression of Rab27A is not significantly impacted.

#### 2.1.1. Rab27B silencing alters CRC cell morphology

**Fig. 2.**
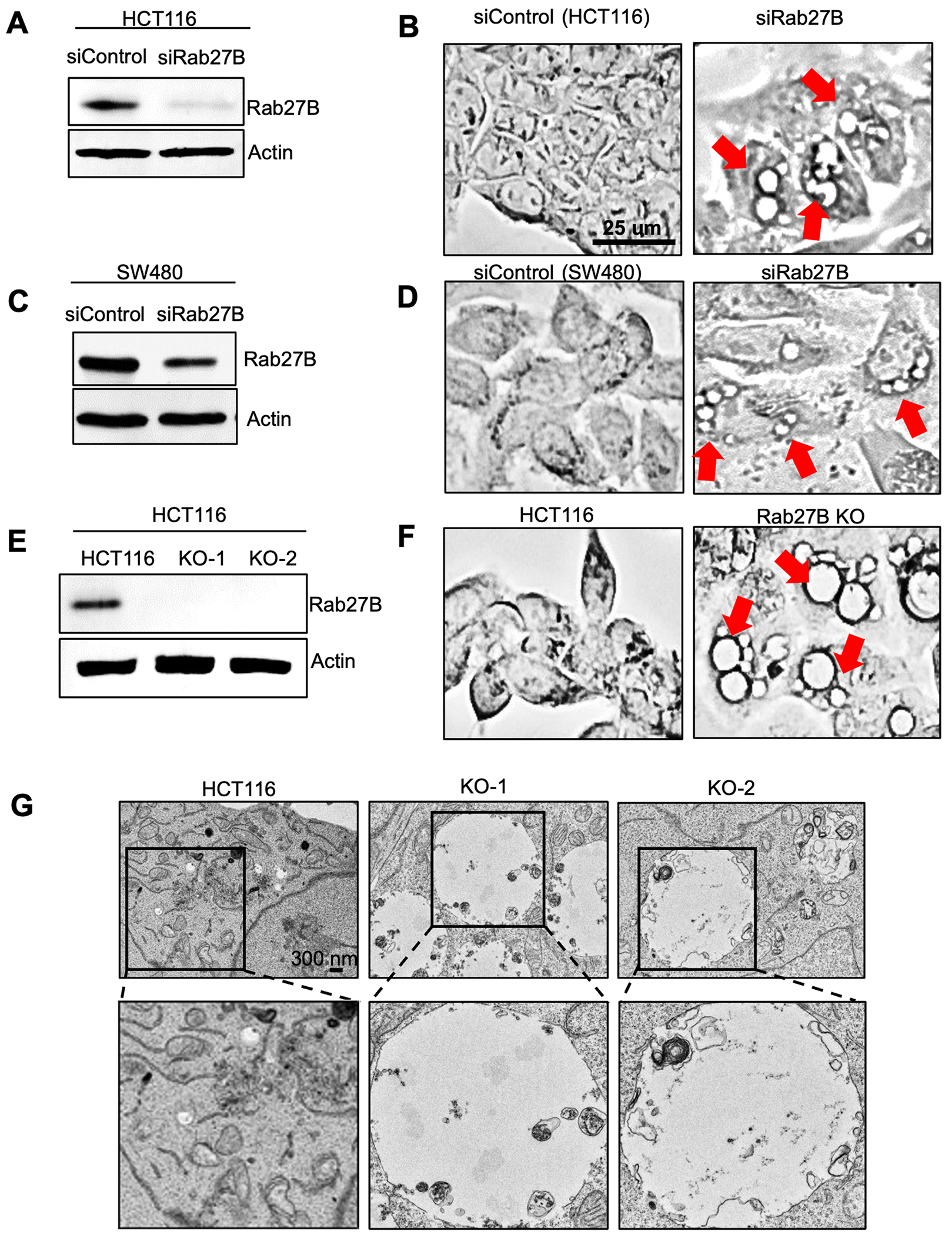
Rab27B suppression results in vesicle formation in CRC cell lines. (A-D) Depletion of Rab27B by siRNA-mediated knockdown resulted in the formation of vesicle-like bodies (indicated by red arrows) in HCT116 (A, B) and SW480 cells (C, D). (E) Rab27B was knocked out by CRISPR/Cas9 in HCT116 cells, resulting in vesicle formation in the KO cells (F). Rab27B knockdown and knockout were verified by western blotting using actin as the loading control. Brightfield images were taken to observe cell morphology. All data presented are representative of four independent experiments. (G) Electron microscopy images of HCT116 WT and Rab27B KO cells. Rab27B KO cells showed an accumulation of autophagosome-like vesicles. Images are representatives of three independent experiments.

To characterize the role of Rab27B in CRC, we performed siRNA-mediated knockdown (KD) of Rab27B in two CRC cell lines, HCT116 and SW480, and observed striking phenotypic differences in the KD cells. The KD cells were approximately double in size and showed an abnormal accumulation of vesicle-like structures (Fig. 2A, B, C, D). We then created CRISPR/Cas9-mediated knockout (KO) of Rab27B in HCT116 cells; Rab27B KO was verified through western blotting in multiple independent clones for Rab27B protein (Figs. S1A and 2E). Consistent with the KD results, the KO cells also showed similar phenotypic changes of increased cell size and accumulation of vesicles (Figs. S1C, S1D, 2F). Rab27A expression was unchanged in the Rab27B KO cells (Figure S1B).

#### 2.1.2. Absence of Rab27B results in increased autophagic vesicles in CRC cells

**Fig. 3.**
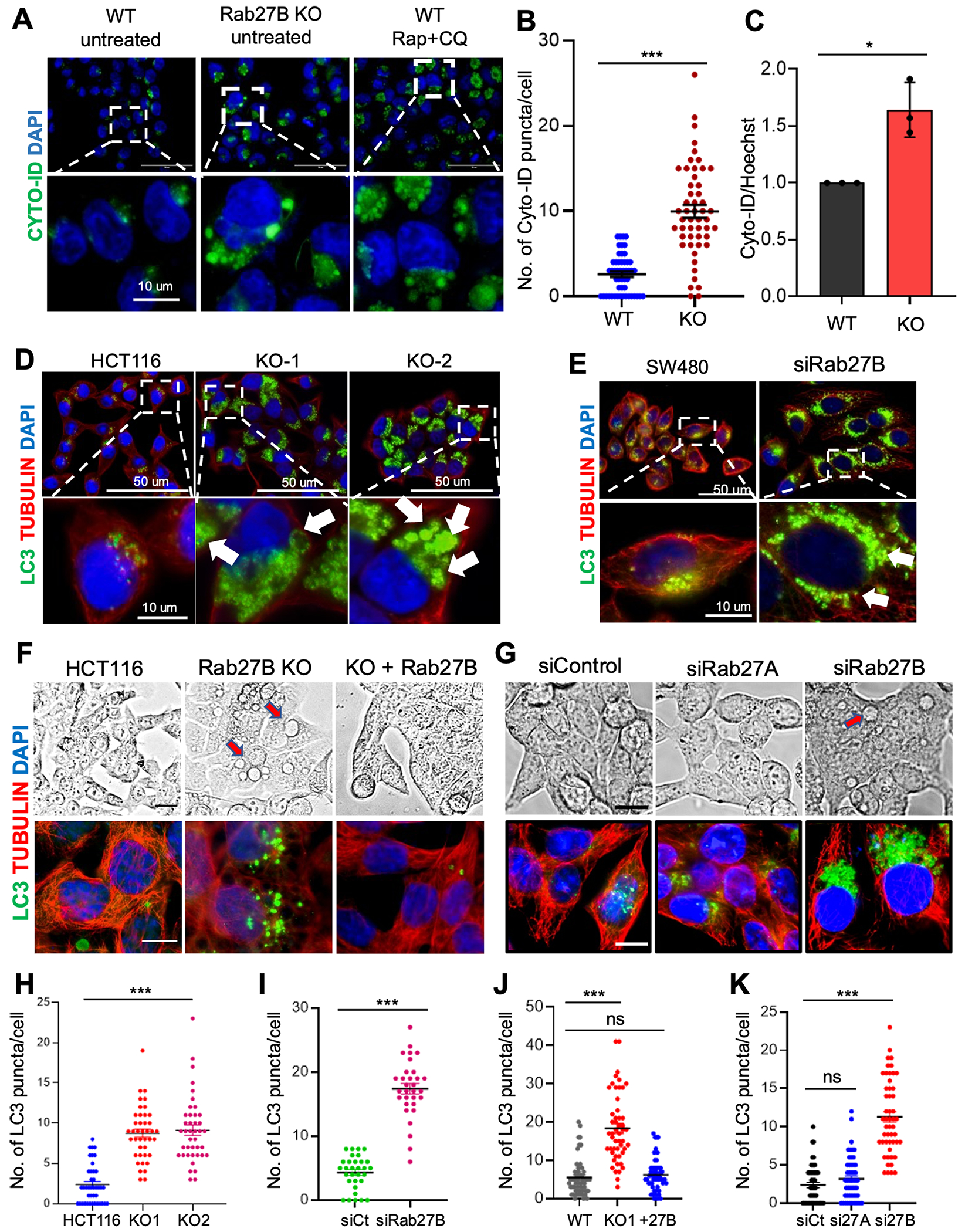
Rab27B KO cells display higher autophagy puncta formation (A) Immunofluorescence images of HCT116 WT and Rab27B KO stained with CYTO-ID dye to label autophagic vesicles and DAPI to stain the nucleus. WT cells treated with rapamycin (0.5 μM) and chloroquine (10 μM) overnight were used as positive control. (B) Comparison of CYTO-ID positive vesicles/cell in WT and KO cells. (C) CYTO-ID absorbance normalized to Hoechst absorbance. (D) HCT116 and Rab27B KO cells were stained with anti-α-tubulin (red signal) and anti-LC3 (green signal) antibodies. Higher magnification images of the area outlined show the presence of punctate LC3-II positive puncta in Rab27B KO cells (white arrows). DAPI (blue) was used to label the nucleus. (E) siRNA-mediated knockdown of Rab27B in SW480 cells was followed by immunofluorescence staining for LC3 and tubulin. (F) Rab27B KO cells were stably transfected with full-length human Rab27B (KO+Rab27B) and stained for LC3 and tubulin immunofluorescence. Bright field images are shown with autophagic vesicles indicated with red arrows. (G) Immunofluorescence staining of HCT116 control, siRab27A, and siRab27B cells with anti-α-tubulin (red signal) and anti-LC3 (green signal) antibodies. Number of LC3 positive vesicles per cell in (H) HCT116 Rab27B KO and (I) siRab27B and siControl (siCt) SW480 cells. (J) Number of LC3 positive vesicles per cell in WT, Rab27B KO, and KO+Rab27B rescue cells. (K) Number of LC3 positive vesicles per cell in siControl, siRab27A, and siRab27B HCT116 cells. Data presented are representative of three independent experiments (*, p ≤ 0.05; **, p ≤ 0.01; ***, p ≤ 0.001).

We used transmission electron microscopy (TEM) to identify and characterize the vesicles observed in Rab27B KO cells. Apparent in the Rab27B KO cells were vesicles that were larger than 500 nm^2^ in area (data not shown), possess double membrane and cytoplasmic contents for degradation, which are characteristics of autophagic vesicles (Fig. 2G) (Klionsky et al., 2016). Identification of autophagic vesicles was done using fluorescent CYTO-ID to selectively label autophagic vesicles at different stages (Klionsky et al., 2016). Untreated HCT116 (WT) cells showed limited autophagy signal and WT cells treated with rapamycin and chloroquine were used as a positive control. Rapamycin induces autophagy and chloroquine blocks autophagic degradation, resulting in an accumulation of autophagic vacuoles. CYTO-ID staining showed a >2-fold increase in CYTO-ID positive vesicles (green) in KO cells compared to untreated WT cells (Figs. 3A and 3B). Similarly, fluorescence intensity of CYTO-ID staining showed a significant increase in the KO cells (Fig. 3C).

Further characterization of this Rab27B KO-dependent accumulation of autophagic vesicles was done using the autophagic marker LC3 protein (Klionsky et al., 2016). LC3 is a soluble protein distributed ubiquitously in mammalian tissues and cultured cells. During autophagy, the 17 kDa cytosolic form of LC3 gets cleaved into a 15 kDa (LC3-I) form, conjugates to phosphatidylethanolamine (LC3-II), gets recruited to autophagosomal membranes, and is recycled by the degradation of autophagosomes (Kabeya et al., 2004). Increased levels of the cleaved LC3-II form indicate an increased presence of autophagosomes. LC3-I and LC3-II can be distinguished in immunofluorescence staining by their distribution; a diffused cytosolic distribution indicates LC3-I, whereas intense puncta-like structures indicate autophagosome localized LC3-II (Barth et al., 2010; Kabeya et al., 2004). We found that the Rab27B KO cells showed a significant increase in LC3-II positive punctate structures (Fig. 3D), with the number of LC3 positive vesicles per cell increase > 3-fold in the KO cells compared to the WT cells (Fig. 3H). To confirm whether this LC3 increase is consistent across other Rab27B-depleted CRC cell lines, Rab27B KD was performed in SW480 and DLD1 cells with a similar increase in LC3-positive vesicles (Figs. 3E, I, S2A, S2B). Next, a rescue experiment was performed by stably transfecting KO cells with full-length Rab27B. Shown in Figs. 3F and J, these cells showed rescue of the autophagy phenotype. Interestingly, knockdown of Rab27A did not change LC3 level or localization in HCT116 cells, indicating that Rab27A does not play any role in autophagy (Figs. 3G, K, S3C). These findings indicate dysregulated autophagy flux in the absence of Rab27B and implicate a possible role of Rab27B in promoting autophagy in CRC.

### 2.2. Rab27B functions in the autophagic pathway

**Fig. 4.**
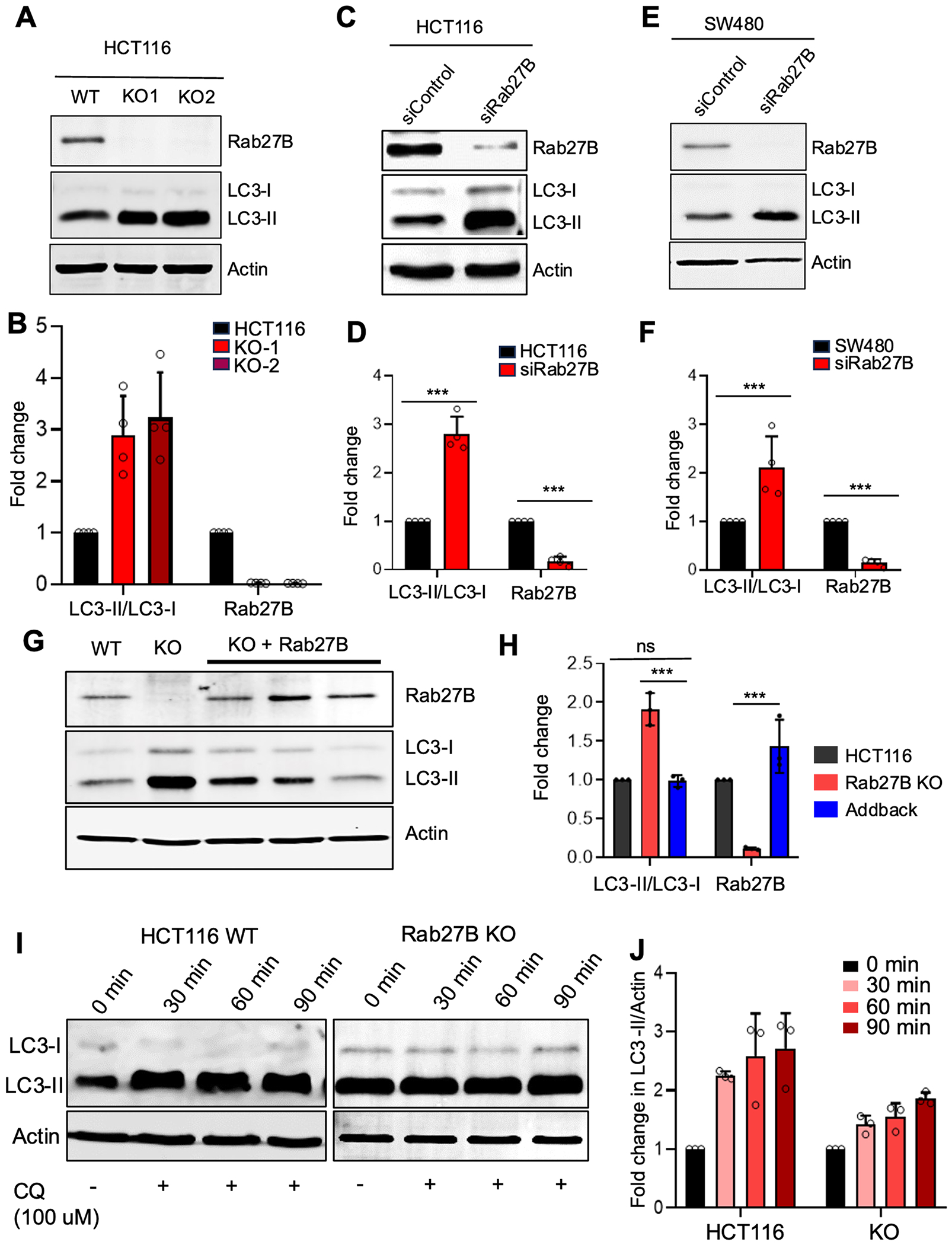
Rab27B depletion increases LC3-II/LC3-I ratio. (A,B) HCT116 WT and Rab27B KO cell lysates were probed for LC3 and Rab27B by western blot, and densitometry analysis of LC3-II/LC3-I ratio and 27B expression using actin was used as the loading control. (C, D, E, F) Western blot analysis of LC3 and Rab27B in Rab27B KD HCT116 and SW480 cells and subsequent densitometry analysis of LC3-II/LC3-I ratio and Rab27B expression. (G) Rab27B KO cells were stably transfected with full-length human Rab27B (KO+Rab27B) and western blot of LC3 and Rab27B in HCT116 WT, Rab27B KO, and 3 clones of Rab27B rescue cells (KO+Rab27B) was performed. (H) LC3-II/LC3-I ratio was calculated by densitometry analysis using ImageJ. (I, J) HCT116 WT and Rab27B KO cells were treated with 100 μM Chloroquine and cells were harvested for western blot analysis 30 min, 60 min and 90 min after CQ treatment. (I) Fold change in LC3-II level after CQ treatment at different time points was calculated by densitometry analysis. Data presented are representative of three independent experiments (*, p ≤ 0.05; **, p ≤ 0.01).

Our results indicate that Rab27B silencing leads to an abnormal accumulation of autophagic vesicles in CRC cells. This accumulation can be caused by an increase in autophagy induction or a block in the degradation of autophagosomes. To identify at which step of autophagy Rab27B functions, we first tested whether there was an increase in the induction of autophagy via the LC3-II/LC3-I ratio. A decrease in the ratio indicates an increase in autophagy induction, whereas an increase in the ratio indicates a block in autophagy degradation (Klionsky et al., 2016). Western blot analysis comparing LC3II/LC3I in HCT116 WT and Rab27B KO cells showed a 2.5- to 3-fold increase in the LC3-II/LC3-I ratio, indicating that a block in autophagy degradation is occurring (Figs. 4A and B). These results were confirmed in siRNA knockdown of Rab27B in HCT116 (Figs. 4C and D) and SW480 (Figs. 4E and F) cells and observed approximately 2.5-fold increase in LC3-II/LC3-I ratio. Furthermore, rescuing Rab27B KO restored the LC3-II/LC3-I level to that observed in WT cells (Fig. 4G).

The difference in the amount of LC3-II in the presence and absence of autophagy inhibitors can be used to examine the turnover of LC3-II through autophagic pathway (Klionsky et al., 2016). If autophagic degradation is occurring normally, a high amount of LC3-II accumulation will occur in response to treatment with degradation inhibitors like chloroquine. However, no difference in LC3-II in the presence of chloroquine may suggest a preexisting block in autophagy at the terminal stage. To confirm that the increase in LC3-II in the KO cells was due to a defect in the autophagy degradation, we treated WT and KO cells with chloroquine over 30–90 minutes. Chloroquine treatment resulted in an immediate increase and accumulation of LC3-II (2–3 fold) in the control WT cells, while the KO cells did not show a significant increase (1.2–1.5 fold) suggesting a block in the autophagy at the degradation stage in the KO cells (Figs. 4I and J).

**Fig. 5.**
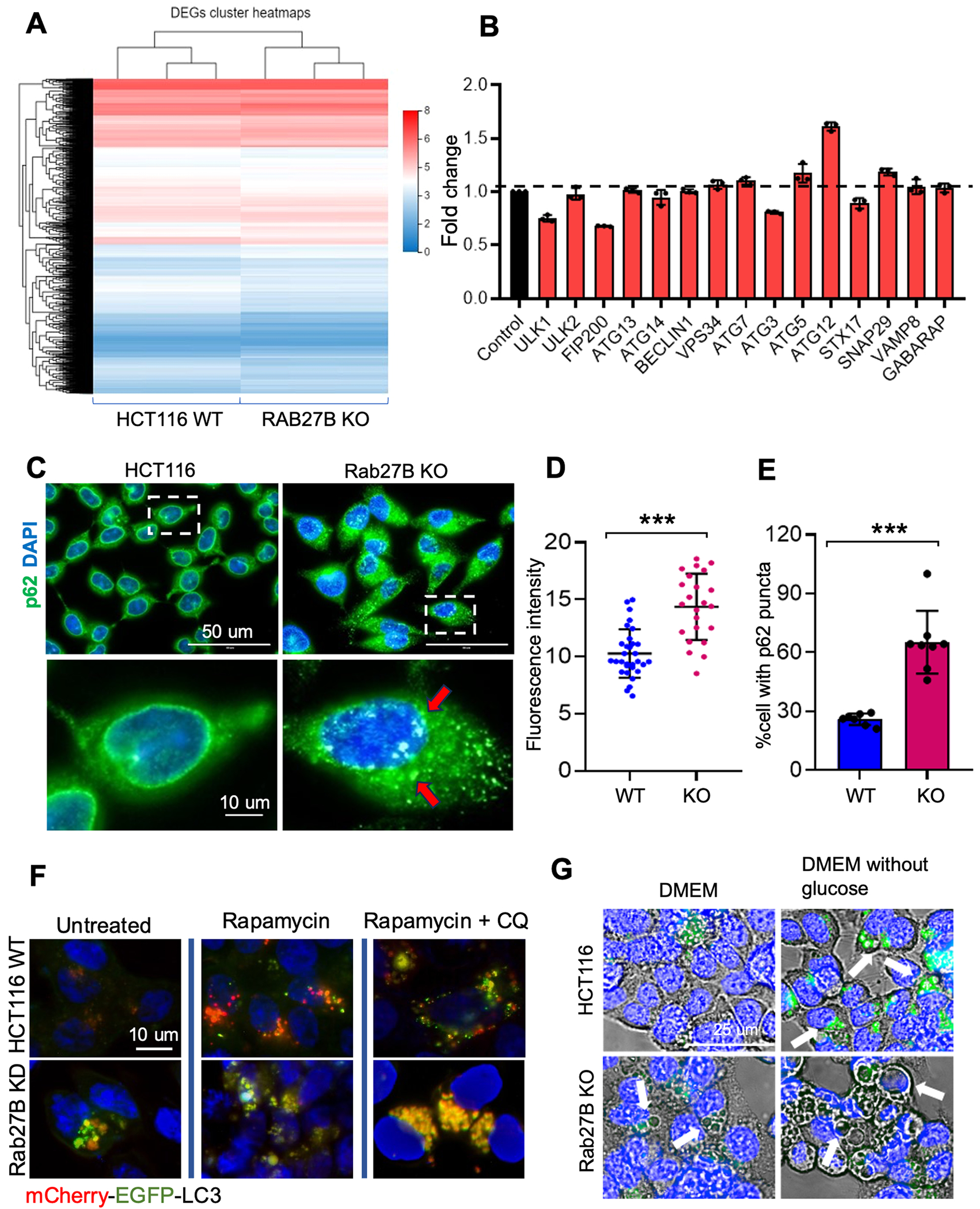
Rab27B deletion impairs autophagy flux. (A) Heatmap of RNA-seq transcriptome analysis of HCT116 WT and Rab27B KO cells. The scale at the right indicates relative expression levels from high (red) to low (blue). (B) Comparison of mRNA expression of different autophagy genes between WT and KO cells obtained from RNA-seq analysis. (C) Immunofluorescence staining of HCT116 and Rab27B KO cells with autophagy marker p62 (green) and DAPI (blue). The KO cells exhibited (D) increased overall p62 fluorescence intensity and a (E) higher percentage of cells with p62 positive vesicles. (F) Rab27B was depleted by siRNA-mediated knockdown in HCT116 cells stably expressing mCherry-EGFP-LC3B. Autophagy flux was monitored by comparing the ratio of mCherry and EGFP double-positive vesicles (yellow) and mCherry-positive (red) vesicles. Cells were treated with rapamycin (0.5 μM for 12 hr) for autophagy induction. Cells treated with rapamycin and chloroquine were used as positive controls. Data presented are representative of three independent experiments (*, p ≤ 0.05; **, p ≤ 0.01; ***, p ≤ 0.001). (G) LysoTracker staining of HCT116 and Rab27B KO cells to label autolysosomes. Hoechst was used for nuclear staining. Cells kept in DMEM without glucose (12 hr) were used as positive controls. Rab27B KO cells showed reduced lysotracker-positive vesicles (indicated by white arrows).

Next, to determine whether there is any dysregulation in the expression of the genes involved in the autophagic pathway in Rab27B KO cells, RNA-seq was performed on HCT116 WT and Rab27B KO cells. The heatmap comparing gene expression between WT and KO cells showed no major differences (Fig. 5A) and expression of autophagy-associated genes ULK1, ULK2, FIP200, ATG13, ATG14, BECLIN1, VPS34, ATG7, ATG3, ATG5, ATG12, STX17, SNAP29, VAMP8, and GABARAP did not show any significant changes in the KO cells (Fig. 5B). Given that Rab27B functions in cellular vesicle trafficking and Rab27B KO CRC cells show an accumulation of LC3-II positive autophagosomes, it is probable that Rab27B functions in the later stages of autophagy (Bjørkøy et al., 2009;Lippai and Low, 2014). To test this, immunofluorescence staining of p62 was performed in HCT116 WT and Rab27B KO cells (Fig. 5C). Rab27B KO cells showed a greater percentage of cells with p62-positive vesicles (60 %) compared to control (20 %) and a 1.5-fold increase in p62 fluorescence intensity/cell (Figs. 5D and E), indicating a block in the autophagic flux in the KO cells.

#### 2.2.1. Rab27B depletion impairs lysosomal fusion during autophagy flux

We used mCherry-EGFP-LC3 reporter to view free autophagosomes and autophagosomes fused with lysosomes (autolysosomes) (Barth et al., 2010; Kimura et al., 2007; Zhang, 2015). LC3-II is incorporated into the inner and outer membranes of expanding and mature autophagosomes, which ultimately fuse with lysosomes for degradation. mCherry-EGFP-LC3 labels two stages of autophagic vesicles: autophagosomes that are double-positive for mCherry and EGFP-fluorescence and autolysosomes that, due to pH sensitivity of GFP, are marked solely by mCherry fluorescence (Figure S3A). To test this, HCT116 cells were stably transfected with mCherry-EGFP-LC3 reporter. Untreated cells show cytosolic distribution of double positive LC3, and treatment with rapamycin and chloroquine to induce and block autophagy flux showed increased autophagosomes (EGFP and mCherry double-positive vesicles) (Figure S3B). Next, siRab27B knockdown was performed in these cells using rapamycin-treated cells for autophagy induction, and rapamycin and chloroquine treated cells as positive controls. Rab27B KD cells showed an increased presence of mCherry-EGFP positive puncta compared to WT cells (Fig. 5F). Rapamycin-treated WT cells showed an increased ratio of mCherry positive red puncta, whereas Rab27B KD cells showed an increased percentage of mCherry-EGFP-positive yellow puncta (Fig. 5F and S3C). This increase in the double-positive mCherry and EGFP-fluorescence in the Rab27B KO cells indicates a block at the fusion stage.

To investigate whether Rab27B promotes autophagosome degradation by directly trafficking autophagosomes to lysosomes or indirectly by affecting lysosomes, we used lysotracker to look at lysosome acidification and distribution. LysoTracker is an acidotropic dye that stains cellular acidic compartments, including lysosomes and autolysosomes (Boya et al., 2003; Byun et al., 2009; Rodriguez-Enriquez et al., 2006). Live cells were stained with LysoTracker and Hoechst dye (nuclear stain) for 5 min prior to immediate imaging, using cells starved without glucose to induce autophagy as a positive control. There was a significant increase in LysoTracker-positive green vesicles in starved WT cells, whereas the vesicles in the starved KO cells remained LysoTracker negative (Fig. 5G). Next, we performed immunofluorescence staining for the lysosomal marker Lamp1 to see any difference in lysosome localization in WT and KO cells. Interestingly, WT cells displayed an even cytosolic distribution of lamp1-positive puncta, whereas in KO cells, these Lamp1-positive puncta appeared to cluster around the autophagic vacuoles (Figure S3D). Taken together, these results indicate that Rab27B is involved in the autophagosome degradation step in the autophagy process in CRC cells.

### 2.3. GTP binding activity of Rab27B is required for autophagy function

**Fig. 6.**
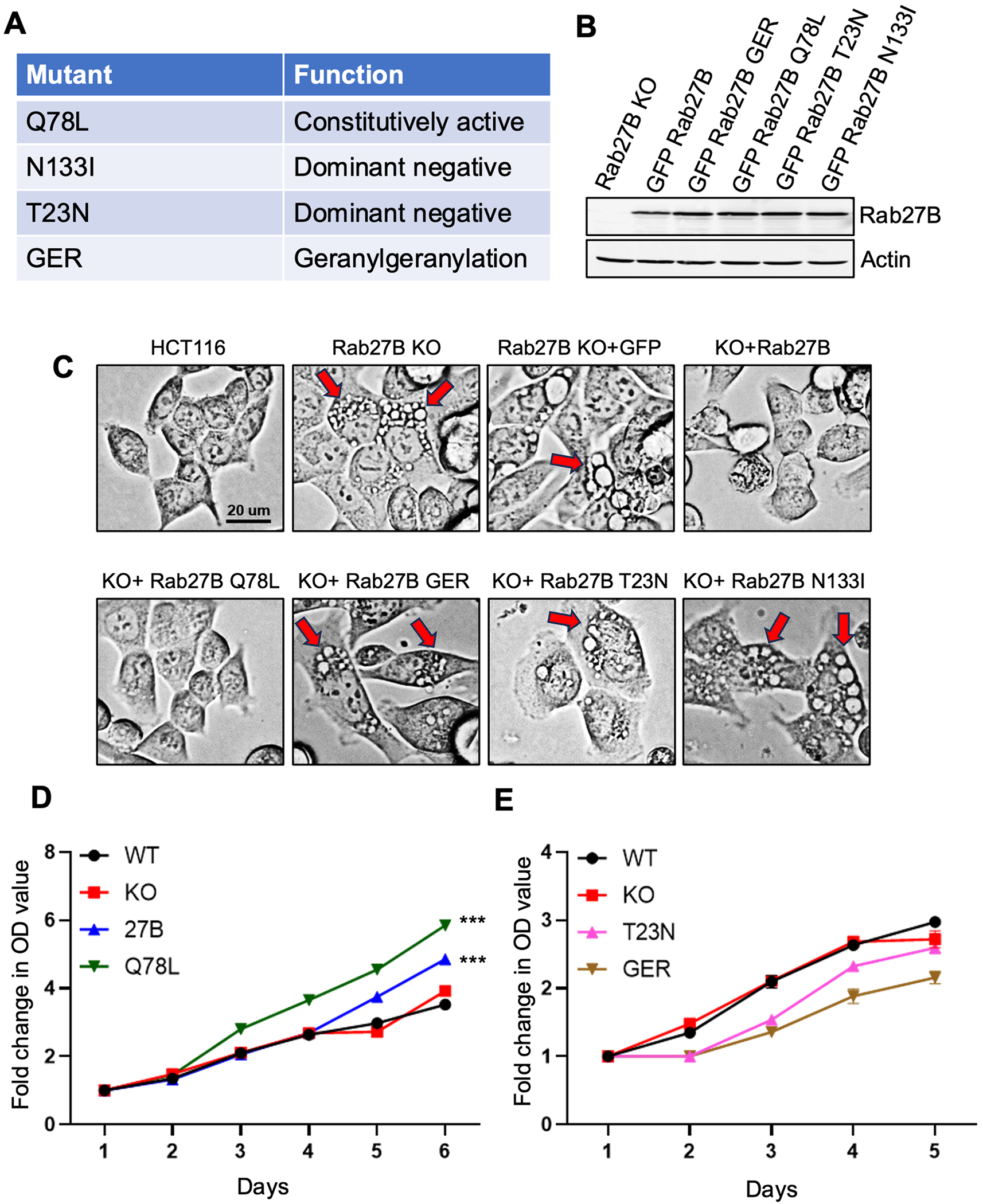
GTP binding of Rab27B is required for autophagy function. (A, B) Rab27B KO cells were stably transfected with GFP-Rab27B fusion constructs indicated as well as WT Rab27B and GFP control. Expression of mutant Rab27B variants by western blot. (C) Brightfield images of indicated Rab27B mutants; red arrows indicate the presence of autophagic vesicle accumulation. (D, E) MTT assays were performed to assess the cell growth of mutant Rab27B cell lines. HCT116 WT and Rab27B KO cells were used as controls. Three independent assays were performed. (*, p ≤ 0.05; **, p ≤ 0.01; ***, p ≤ 0.001).

Rab27B is a GTPase and functions by switching between active GTP bound and inactive GDP bound states. To determine whether the GTP binding function of Rab27B is required for the autophagy, we utilized vectors containing mutant forms of GFP-tagged Rab27B as follows: constitutively active GTP mutant Q78L, dominant negative GDP mutant N133I, and T23N, and geranylgeranylation mutant GER along with WT form of Rab27B (Fig. 6A). These vectors were stably transfected into Rab27B KO HCT116 cells to generated stable cell lines (Fig. 6B). Brightfield images showed restoration of autophagy phenotype only in the WT and constitutively active Q78L Rab27B expressing cells (Fig. 6C). KO cell lines expressing dominant negative Rab27B-T23N and Rab27B-N133I, or geranylgeranylation mutant Rab27B-GER showed accumulation of autophagic vesicles. This result indicates that GTP binding of Rab27B is necessary for the autophagy function. We also compared the proliferation rate of the mutant Rab27B expressing cell lines by MTT assay. Interestingly, constitutively active GTP mutant Q78L cells showed significantly increased proliferation compared to WT or KO cells (Fig. 6D), whereas cells expressing Rab27B-T23N or Rab27B-GER did not impact growth significantly (Fig. 6E).

### 2.4. Rab27B depletion inhibits in vitro 3D growth and in vivo tumorigenesis

Autophagy in cancer has been shown to promote tumor growth and cancer cell survival, and inhibiting autophagy can attenuate tumor cell growth. Our results show that silencing Rab27B leads to autophagic dysregulation in cancer cells, suggesting that Rab27B depletion might also affect cancer cell growth. To determine whether the KO cells show any growth difference under 2D conditions, MTT and cell counting assays were performed over five days with comparable growth rates observed between WT and Rab27B KO cells (Figures S4A and S4B). Similarly, colony formation assays did not show any change in colony forming ability of the Rab27B KO cells (Figures S4C and S4D). We also performed invasion and migration assays, but did not observe any significant difference between WT and Rab27B KO cells (Figures S4E, S4F, S4G, S4H)

**Fig. 7.**
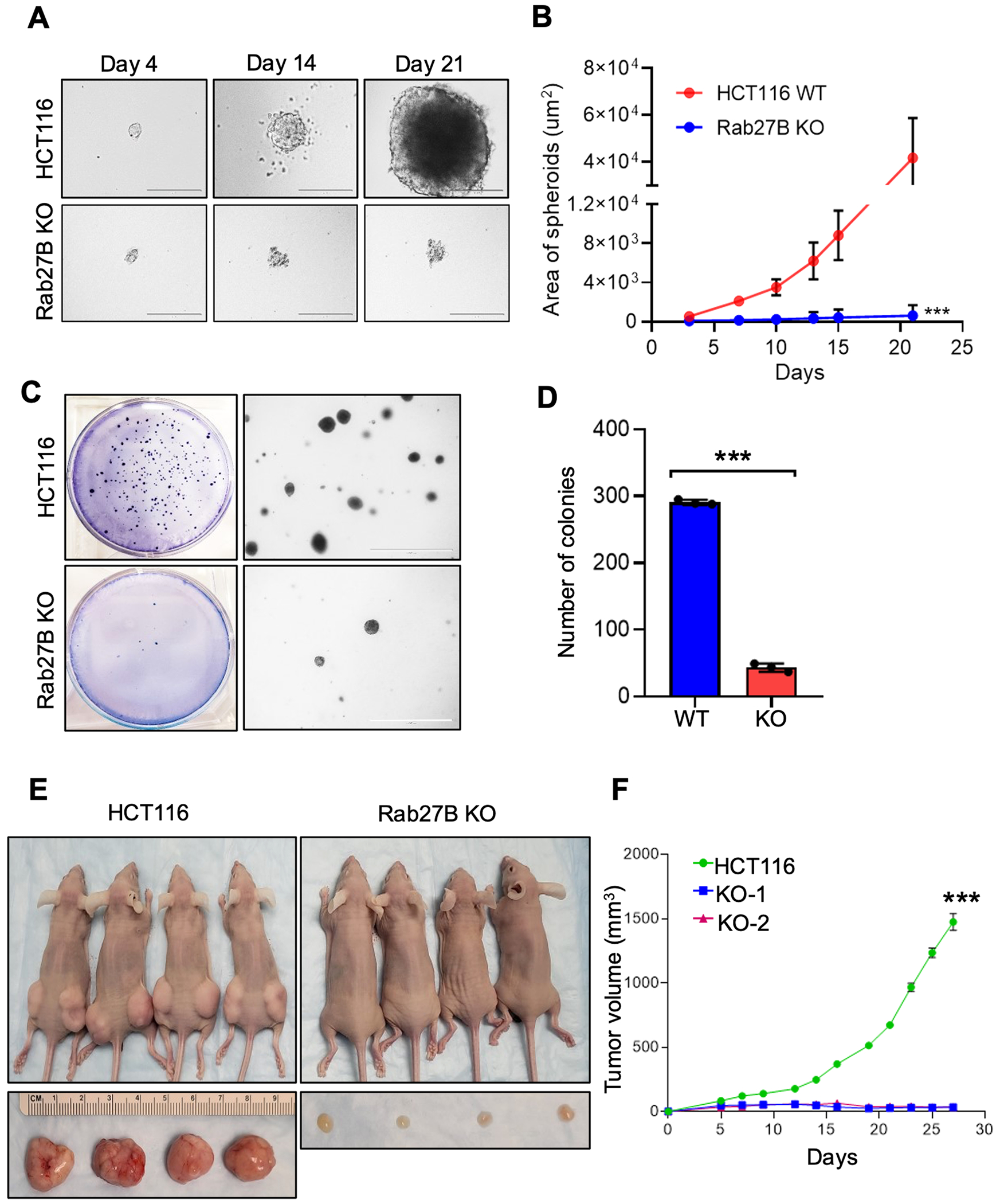
Rab27B depletion inhibits in vitro 3D growth and in vivo tumorigenesis. (A) Brightfield images of spheroids formed by HCT116 WT and Rab27B KO cells at day 4, day 14 and day 21. (B) The area of the spheroids was assayed using ImageJ software. (C, D) HCT116 WT and Rab27B KO cells seeded at 5000 cells/well density for growth in soft agar and were stained with 0.1 % crystal violet to visualize and count colonies. The results shown are representative of 3 independent experiments. (E, F) HCT116 WT and two separate Rab27B KO clones were injected (1 million cells) subcutaneously into the flanks of 5–6 weeks athymic nude mice (n=8 tumors/group). Tumors were measured 3x/week. Representative images of mice and harvested tumors from control and Rab27B KO groups 28 days post injection, along with tumor volumes are shown. Values given are expressed as mean ± SEM (n=8 per group). Spheroids and soft agar colonies were counted using a brightfield microscope. (*, p ≤ 0.05; **, p ≤ 0.01; ***, p ≤ 0.001).

Recent research has shown that Rab27B can play a role in cancer stem cell growth and spheroid formation (Cheng et al., 2019; Meneses et al., 2023). To evaluate the growth of Rab27B KO cells in 3D culture condition, HCT116 (WT) and Rab27B KO cells were plated at 3–4 cells/well concentration in ultra-low adherence 96-well plates, and spheroid formation and size was monitored over 3 weeks (Fig. 7A). Interestingly, HCT116 WT cells expanded into large spheroids, while the majority of the KO cells failed to grow spheroids (Fig. 7B). When assaying anchorage-independent growth by soft agar colony formation, Rab27B KO cells showed a 94 % reduction in colony number (Figs. 7C and D). To determine if Rab27B deletion affects CRC cell growth *in vivo*, subcutaneous xenograft tumor growth assays were performed. 5–6 weeks old nude mice were injected with HCT116 WT and Rab27B KO clones (1 million cells/injection), and tumor volume was measured 3x/week. Similar to *in vitro* 3D growth results, Rab27B KO cells failed to grow xenograft tumors *in vivo* (Figs. 7E and F).

### 2.5. Rab27B KO cells are susceptible to starvation

Cancer cells deal with several stresses like scarcity of nutrition, hypoxia, immune response under *in vivo* conditions. As autophagy contributes to resistance against these stresses, we hypothesized that the autophagy-defective Rab27B KO cells should be more susceptible to these stresses. To test this, we compared the growth and survival of HCT116 and Rab27B KO cells exposed to different starvation conditions for 24 hr as follows: DMEM (4.5 g/l glucose), DMEM (1 g/l glucose), DMEM (0 g/l glucose); cells grown in DMEM (4.5 g/L glucose containing 10 % FBS) was used as a positive control. Shown in Figures S5A and S5B, Rab27B KO cells had significantly reduced survival under nutrient stress-related serum-free conditions compared to WT cells. Interestingly, we observed an increase in the Rab27B protein levels following starvation in WT cells (Figure S5C), suggesting increased expression of Rab27B may promote autophagy under stress. This implies that Rab27B assists in tumor cell survival under conditions of stress (starvation) that are more representative of the tumor microenvironment.

## 3. Discussion

In this study, we have identified Rab27B as a critical component of the regulation of autophagy in CRC. Silencing or deletion of Rab27B led to a defect in autophagosome degradation, resulting in an abnormal accumulation and size of autophagosomes. Our data indicates that fusion of autophagosomes and lysosomes is deficient in the Rab27B depleted cells resulting in defective autophagy flux. This functional impact of Rab27B in the autophagy process depends on the GTP binding and geranylgeranylation of Rab27B. While Rab27B deficient cells grow under conventional 2D conditions, growth under anchorage-free 3D conditions and *in vivo* was dramatically attenuated. Together, these results show the importance of Rab27B in regulating autophagy and identify Rab27B as a novel tumor promoter, highlighting the therapeutic potential of Rab27B in CRC.

Rab27A and Rab27B are homolog Rabs that are major regulators of vesicle fusion and secretion in various intracellular trafficking processes (Li et al., 2018; Ostrowski et al., 2010). Despite having a structural similarity of 70 %, Rab27A and Rab27B show diverse expression and functions in different tissues. Rab27A is overexpressed in glioblastoma, prostate, pancreas, melanoma, and thyroid cancer, whereas Rab27B shows high expression in breast, cervical, kidney, lung, colon, and stomach cancers (Tang et al., 2017). Our previous results show that Rab27B can be overexpressed through post-transcription regulation. The RNA-binding protein HuR regulates Rab27B expression post-transcriptionally by binding the 3’ UTR region of Rab27B mRNA, resulting in stabilization and subsequent protein overexpression (Xiao et al., 2022)(in submission). Interestingly, HuR does not appear to regulate Rab27A expression, which agrees with the observation that Rab27A is not overexpressed in CRC. Here, we show that Rab27B is overexpressed in CRC tumors compared to normal colon, consistent with prior observations showing Rab27B overexpression in CRC correlates with poor prognosis and survival (Bao et al., 2014; Cheng et al., 2019).

Rab27A and Rab27B not only show diversity in their expression but also their functions. Studies have shown that Rab27A and Rab27B can perform different functions attributed to their interaction with various Rab27 effector proteins (Fukuda, 2013; Ostrowski et al., 2010). Both of these Rabs have been implicated in the proliferation, invasion, and progression of multiple cancers. In non-small cell lung cancer (NSCLC), Rab27B can drive cancer stem cell (CSC)-like phenotype through small extracellular vesicle (sEV) secretion (Meneses et al., 2023). Through its ability to increase sEV secretion through Rab27B overexpression, NSCLC CSCs can promote spheroid growth, clonal expansion, and invasion in bulk cancer cells. In myeloid malignancies, Rab27B is overexpressed and promotes cancer progression through NRAS palmitoylation and trafficking (Ren et al., 2023). Through its ability to promote enhanced sEV secretion in CRC stem cells, Rab27B can influence tumorigenicity and progression through trafficking increased mir-146a-5p through sEVs (Cheng et al., 2019). Here, we demonstrate a novel function of Rab27B in regulating the autophagy process in CRC. Rab27B silencing both by siRNA-mediated knockdown and CRISPR/cas9 knockout resulted in the accumulation of autophagosome-like vesicles in CRC cells. Similar approaches used to deplete Rab27A did not result in any autophagy defect (in preparation). These results indicate that Rab27A and Rab27B may have different functions in CRC cells, apart from sEV secretion.

The accumulation of autophagosomes in Rab27B deficient cells can be a result from two distinct events - an increase in autophagosome formation resulting from enhanced initiation events, or a defect in autophagosome turnover resulting from a block in the later stages of autophagy. RNA-seq data did not indicate any major changes in autophagy gene expression involved in the initial stages of autophagy or formation of autophagosomes, and monitoring autophagy flux did not show any change in the autophagy initiation in the Rab27B-depleted cells as well. Additionally, the late autophagy marker p62 revealed an accumulation of p62-positive puncta in Rab27B knockout cells. These results indicate that autophagy defect in Rab27B KO cells occurs in the degradation step of the autophagosomes. These results are consistent with prior work showing that Rab27B KD resulted in accumulation of LC3-positive vesicles with defects in autophagy flux and accumulation of autophagosomes in neuroblastoma cells, and Rab27A/B double KO mice showed an increase in autophagosomes and lysosomes in the lacrimal gland (Chiang et al., 2011; Underwood et al., 2020) indicating possible impairment in autophagosome-lysosome fusion. To monitor autophagy turnover, we used the EGFP/mCherry-tagged LC3 expression system and show that Rab27B depletion resulted in EGFP and mCherry double-positive vesicles, indicating these vesicles were not autolysosomes. This defect in turnover can occur at the fusion stage (autophagosome-lysosome fusion) or inefficient cargo degradation once fusion has occurred. Using LysoTracker, our results indicate that autophagic vesicles in Rab27B KO cells are not LysoTracker positive in response to induction of autophagy. Together these results show that Rab27B depletion impairs autophagosome-lysosome fusion, implicating that Rab27B functions at the fusion stage of autophagy.

Rab27B functions through GTPase activity and switches between active GTP bound and inactive GDP bound conformations. Rab27B also undergoes post-translational modification by geranylgeranylation, which serves as a membrane anchoring mechanism. Prior work in ERpositive breast cancer has shown functional necessity of Rab27B’s GTPase activity in facilitating tumor cell growth, invasion, and metastasis through a GTP-binding and geranyl-geranyl dependent manner (Hendrix et al., 2010). Here, in a Rab27B KO background, we demonstrate rescue of the autophagy-stalled phenotype through expression of Rab27B WT and constitutively active Q78L mutant. GTP binding mutants T23N and N133I, as well as geranylgeranyl mutant GER, did not show any recovery of the autophagy dysregulation. Overall, these results indicate that the autophagy function of Rab27B is dependent on its GTP binding and geranylgeranyl modification.

Interestingly, Rab27B deletion does not affect the proliferation or colony formation ability of CRC cells *in vitro* in 2D culture, we do observe a striking growth deficiency under *in vitro* 3D culture conditions as well as with *in vivo* tumor growth. Rab27B KO cells failed to grow spheroids and showed a 94 % reduction in soft agar colony formation assay. These results highlight the importance of Rab27B in anchorage-independent growth, which is characteristic of cancer stem cells. This is consistent with results showing Rab27B being important component in the growth of NSCLC stem-like cells (Meneses et al., 2023). Cancer stem cells require high autophagy flux, so defective autophagy could drastically affect these cells (Chen and Debnath, 2010). One area where this manifests with Rab27B loss is during *in vivo* tumor growth, with Rab27B KO cells growing drastically smaller tumors than WT cells. Consequently, when autophagy is impaired, CRC cells show a similar significant reduction in xenograft tumor growth *in vivo* (Lauzier et al., 2019). This growth phenotype most likely reflects a higher requirement for autophagy under *in vivo* condition. Even though Rab27B KO cells do not show growth reduction in 2D growth *in vitro*, we did observe enhanced growth in Rab27B KO cells expressing the constitutively active Rab27B-Q78L mutant. Further efforts are underway characterizing this oncogenic potential of Rab27B.

The results presented here identify a potential mechanism by which Rab27B facilitates autophagy and emphasizes the need for Rab27B-mediated autophagy under stress-associated growth conditions seen in CRC (i.e., anchorage-free cell and *in vivo* tumor growth). While our results demonstrate that Rab27B is involved in the autophagic process and promotes autophagic flux, the mechanism of how Rab27B facilitates this role remains to be further elucidated. Based on its established role in vesicle trafficking, we hypothesize that Rab27B serves to traffic autophagosome and lysosome interaction and fusion. In support of this, Rab27B has been identified to bind with key protein complexes involved in the autophagy process (Oughtred et al., 2021). Of interest, the lysosome integral membrane protein-2 (LIMP2) has been detected to interact with Rab27B (Liu et al., 2023), suggesting a putative interaction connecting Rab27B to lysosomes. To date, eleven Rab27-specific effectors have been identified (Oughtred et al., 2021). Based on this, we predict that Rab27B may interact with different effector proteins to regulate the autophagy process or sEV secretion. This underscores the promising therapeutic potential of targeting Rab27B and impacting both autophagy and sEV secretion necessary for tumor progression. Future investigations will focus on discovering specific inhibitors against Rab27B for CRC prevention and treatment.

## Supplementary Material

Supplementary Material

## Data Availability

Data will be made available on request.

## Appendix A. Supporting information

Supplementary data associated with this article can be found in the online version at doi:10.1016/j.biocel.2024.106693.
