# Supplementary material for: Regulation of autophagy by Rab27B in colorectal cancer
Source: Int J Biochem Cell Biol. Author manuscript; Available in PMC 2026 Aug 18. (PMC13485073; doi:10.1016/j.biocel.2024.106693)
Supplement: Supplementary file 1 [file NIHMS2199414-supplement-Supplementary_Material.pdf]

SUPPLEMENTAL INFORMATION

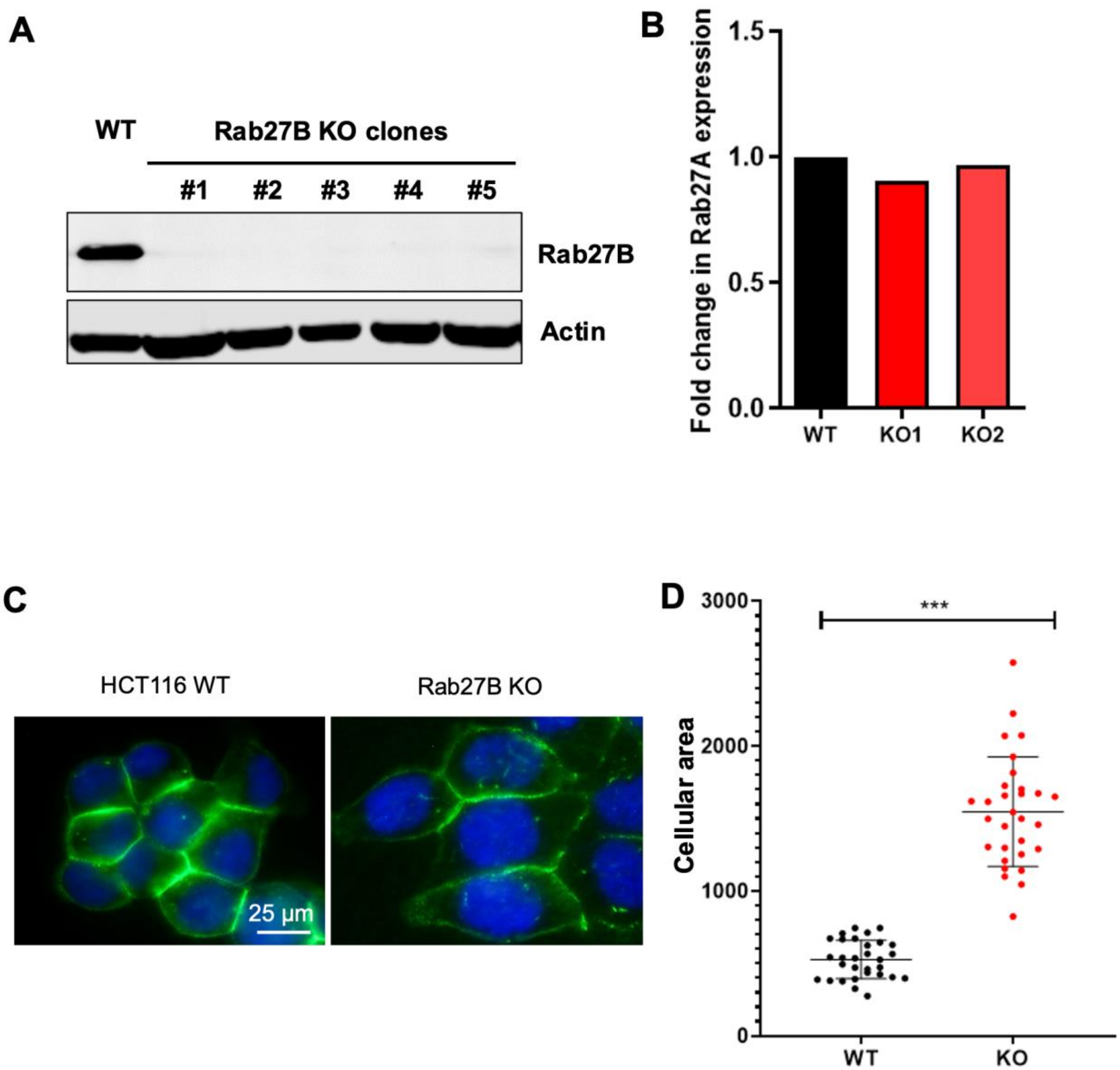

Figure S1. *Establishment of Rab27B KO HCT116 cells.* ***(A)*** *Individual Rab27B KO clones were isolated and screened by WB for the loss of Rab27B protein. Actin was used as loading control.* ***(B)*** *qPCR was performed to measure Rab27A mRNA expression in Rab27B KO cells.* ***(C)*** *Immunofluorescence staining of HCT116 WT and Rab27B KO cells for E-Cadherin and Dapi to visualize cell size.* ***(D)*** *Area of the cells were calculated by using ImageJ. The data shown are representative of three independent experiments. (*, p ≤ 0.05; **, p ≤ 0.01; ***, p ≤ 0.001).*

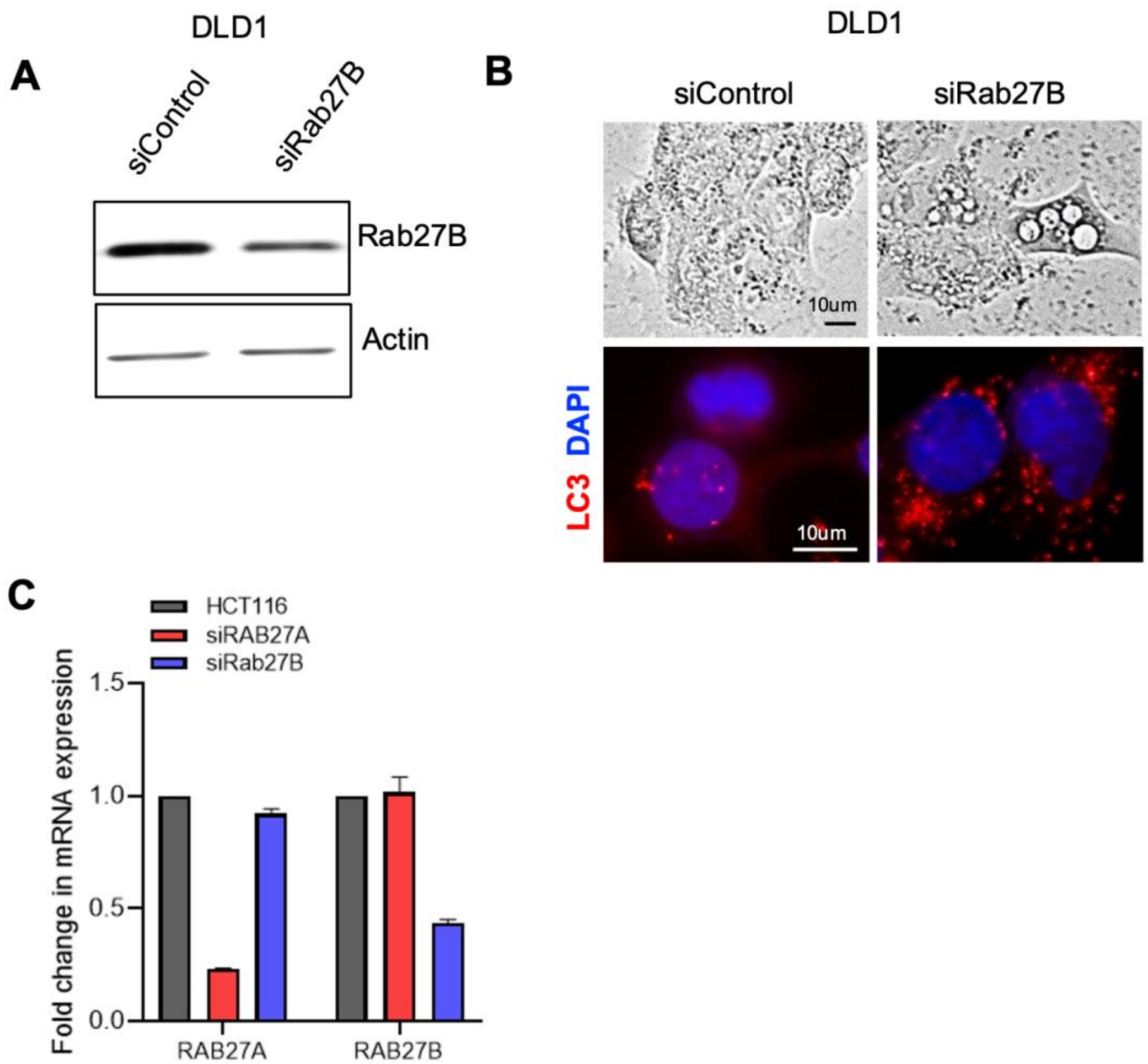

Figure S2. *LC3 immunostaining of Rab27B knockdown DLD1 cells.* ***(A)*** *Western blot of Rab27B KD in DLD1 cells.* ***(B)*** *Brightfield and immunofluorescence images of LC3 positive vesicles (red) in DLD1 cells after Rab27B KD.* ***(C)*** *Expression of Rab27A and Rab27B mRNA by qPCR after respective siRNA knockdown was was performed in HCT116 cells. The data shown are representative of three independent experiments. (*, p ≤ 0.05; **, p ≤ 0.01; ***, p ≤ 0.001).*

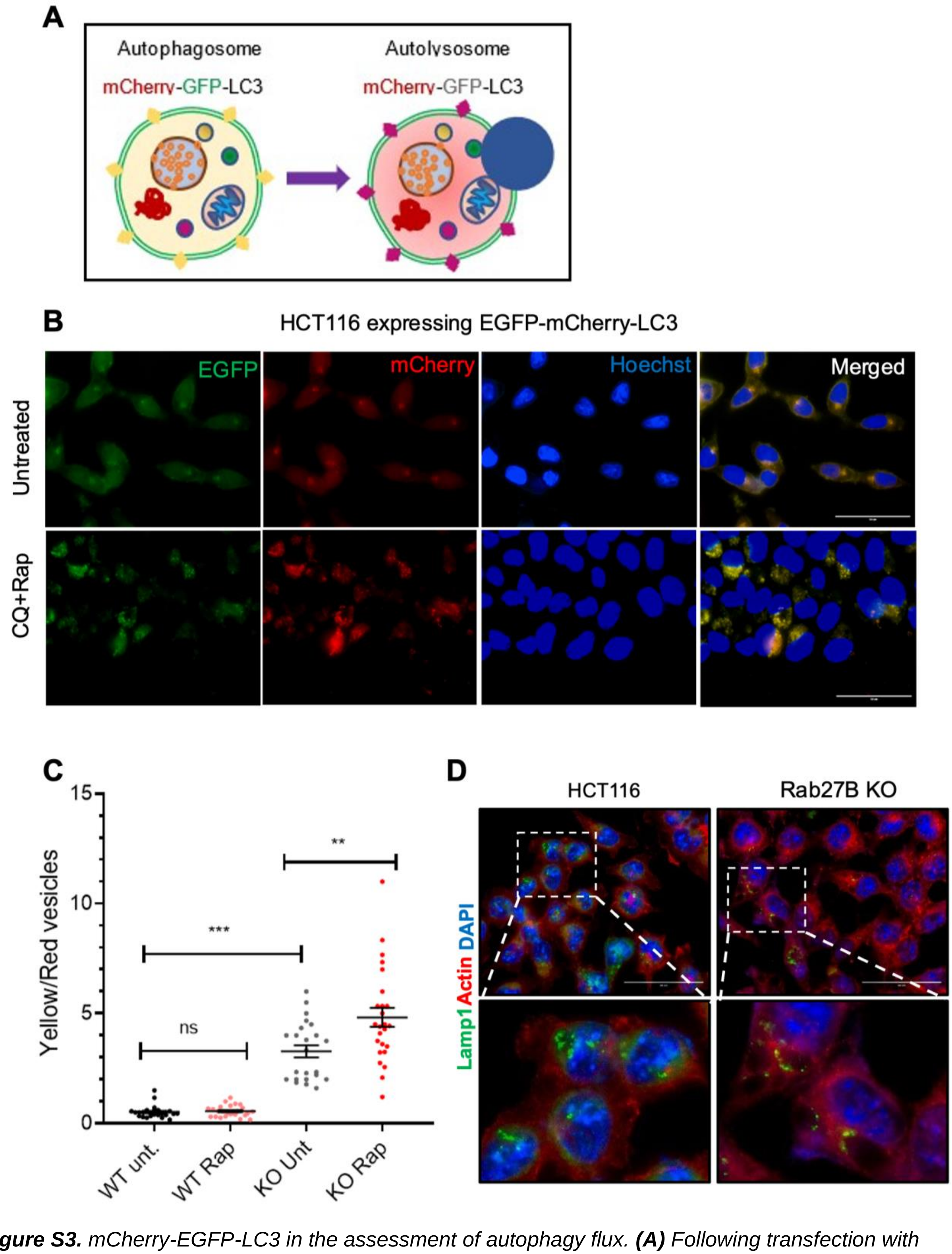

Figure S3. *mCherry-EGFP-LC3 in the assessment of autophagy flux.* ***(A)*** *Following transfection with mCherry-GFP-LC3 reporter plasmid, mCherry-GFP-LC3 localizes to autophagosome membranes. Autophagosomes are double positive for mCherry and EGFP (indicated by yellow puncta). Autolysosomes are positive only for mCherry due to the degradation of EGFP following lysosomal fusion (indicated by red puncta).* ***(B)*** *HCT116 stably expressing EGFP-mCherry -LC3 fusion protein was created. Untreated control cells show cytosolic LC3 (yellow). Cells treated with rapamycin (0.5 mm) and*

chloroquine (10 mm) show the formation of yellow and red autophagic vesicles. ***(C)*** *Autophagy flux was monitored by comparing the ratio of mCherry and EGFP double-positive vesicles (yellow) and mCherry-positive (red) vesicles. Cells treated with rapamycin (0.5mm for 12h) were used as positive controls. (D) Immunofluorescence staining of HCT116 WT and Rb27B KO cells for lysosomal marker Lamp1 (green signal), actin (red signal) and Dapi (blue signal). Data presented are representative of three independent experiments. (*, $p \le 0.05$; **, $p \le 0.01$; ***, $p \le 0.001$).*

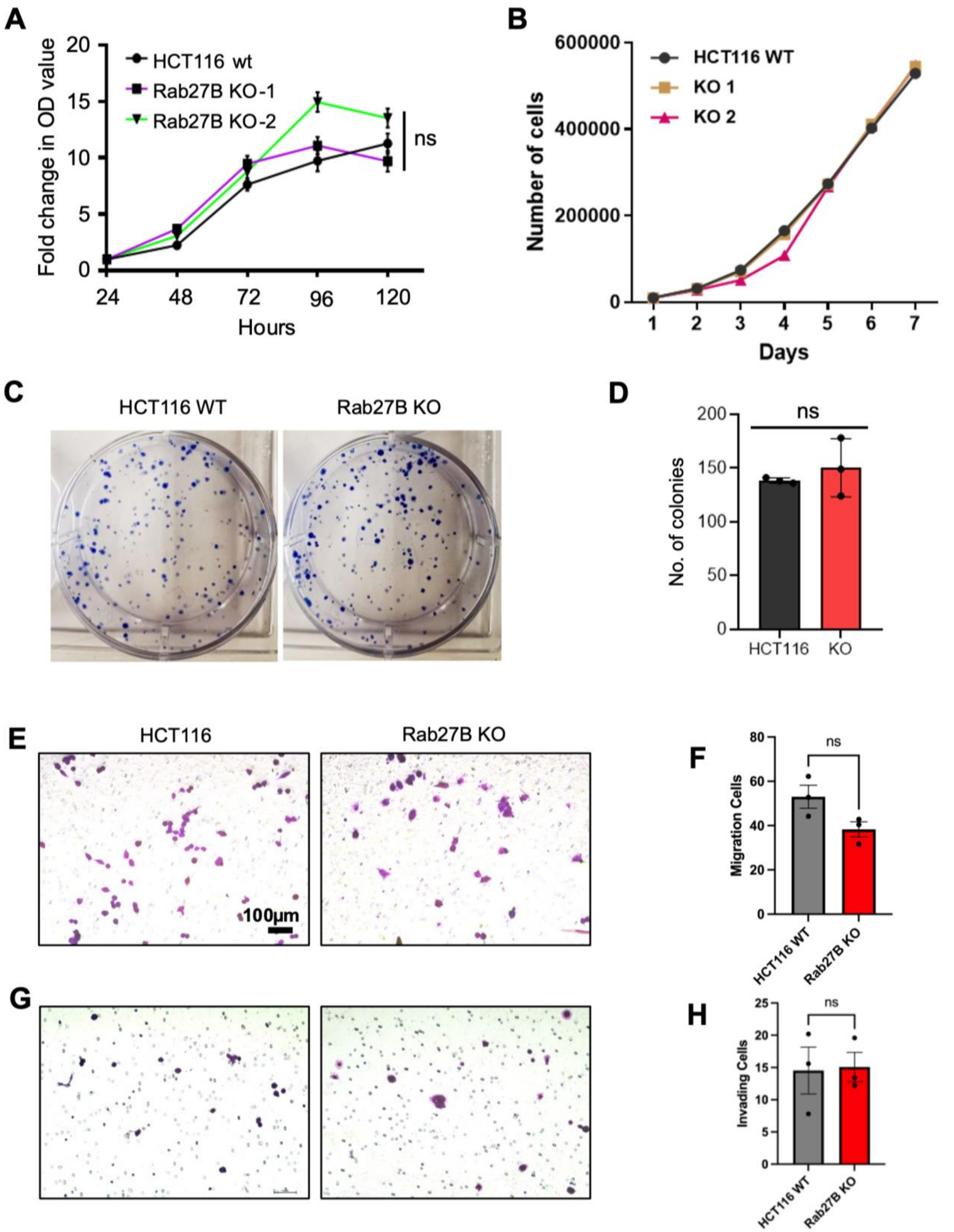

Figure S4. *Rab27B KO cells do not show any change in proliferation in 2D conditions.* ***(A)*** *The proliferation of HCT116 and Rab27B KO cells grown in standard growth conditions (DMEM with 10% FBS) was measured by MTT assay. Fold change was calculated by normalizing the optical density (OD) values of each day to the initial OD values recorded on day 1.* ***(B)*** *HCT116 and Rab27B KO cells were grown in 6*

well plates and cell counting was done every day for seven days. ***(C)*** *Colony formation assays were performed to compare the colony-forming ability of HCT116 and Rab27B KO cells.* ***(D)*** *The number of colonies was counted under the microscope.* ***(E)*** *Representative images transwell migration assay of HCT116 WT and Rab27B KO cells.* ***(F)*** *Quantification of the average number fo migrating cells/ field.* ***(G)*** *Transwell invasion assay of HCT116 and Rab27B KO cells using Matrigel coated transwell (Corning).* ***(H)*** *Quantidication of the average number of invading cells/ field. Experiments were done in triplicates and repeated three times. The result is shown as mean ± SEM. (*, $p \le 0.05$; **, $p \le 0.01$; ***, $p \le 0.001$).*

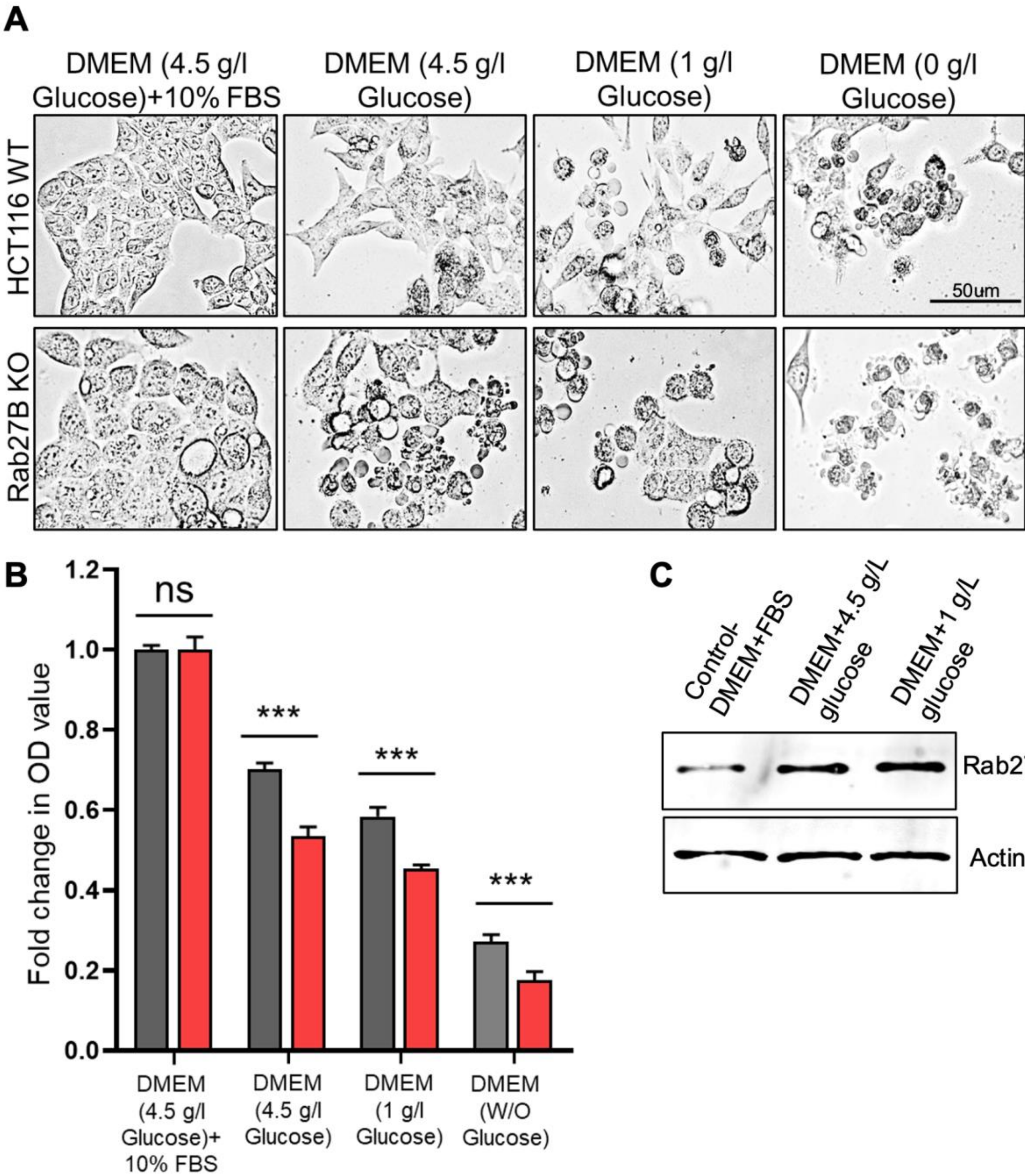

Figure S5. *Rab27B KO cells show reduced survival percentage following starvation.* ***(A)*** *HCT116 and KO cells were grown in 96-well plates containing DMEM with 10% FBS for 48 hr, followed by serum starvation in only DMEM (4.5 g/l glucose), DMEM with low glucose (1g/l glucose) and DMEM without any glucose for 24 hours.* ***(B)*** *Cell survival was measured by MTT assay normalized to the initial OD value before starvation. Cells grown in DMEM (4.5 g/l glucose) supplemented with 10% FBS were used as positive controls.* ***(C)*** *Western blotting shows an increase in Rab27B protein level following starvation in HCT116 WT cells. Actin was used as the loading control. Experiments were repeated three times. The results are shown as mean ± SEM. (*, $p \leq 0.05$; **, $p \leq 0.01$; ***, $p \leq 0.001$).*
